# Use of Digitalisation and Machine Learning Techniques in Therapeutic Intervention at Early Ages: Supervised and Unsupervised Analysis

**DOI:** 10.3390/children11040381

**Published:** 2024-03-22

**Authors:** María Consuelo Sáiz-Manzanares, Almudena Solórzano Mulas, María Camino Escolar-Llamazares, Francisco Alcantud Marín, Sandra Rodríguez-Arribas, Rut Velasco-Saiz

**Affiliations:** 1 DATAHES Research Group, Consolidated Research Unit Nº. 348, Departamento de Ciencias de la Salud, Facultad de Ciencias de la Salud, Universidad de Burgos, 09001 Burgos, Spain; cescolar@ubu.es; 2 Unidad de Atención Temprana, ASPACE Salamanca, 37185 Villamayor de Armuña, Spain; almumj@hotmail.com; 3 Department of Developmental and Educational Psychology, Universitat de València, 46010 València, Spain; francisco.alcantud@uv.es; 4 BEST-AI Research Group, Departamento de Ingeniería Informática, Escuela Politécnica Superior, Universidad de Burgos, 09006 Burgos, Spain; srarribas@ubu.es; 5 Facultad de Ciencias de la Salud, Universidad de Burgos, 09001 Burgos, Spain; rvx1008@alu.ubu.es

**Keywords:** early care, digitalization, machine learning

## Abstract

Advances in technology and artificial intelligence (smart healthcare) open up a range of possibilities for precision intervention in the field of health sciences. The objectives of this study were to analyse the functionality of using supervised (prediction and classification) and unsupervised (clustering) machine learning techniques to analyse results related to the development of functional skills in patients at developmental ages of 0–6 years. We worked with a sample of 113 patients, of whom 49 were cared for in a specific centre for people with motor impairments (Group 1) and 64 were cared for in a specific early care programme for patients with different impairments (Group 2). The results indicated that in Group 1, chronological age predicted the development of functional skills at 85% and in Group 2 at 65%. The classification variable detected was functional development in the upper extremities. Two clusters were detected within each group that allowed us to determine the patterns of functional development in each patient with respect to functional skills. The use of smart healthcare resources has a promising future in the field of early care. However, data recording in web applications needs to be planned, and the automation of results through machine learning techniques is required.

## 1. Introduction

Child development can be understood as an interaction (biological–genetic) between the organism and the environment. When a child is affected by a neurodevelopmental disorder, early intervention programmes are implemented to prevent socio-familial disruption, increase appropriate interaction between the child and the family, and improve family functioning. Intervention helps families adjust to the new situation and provides necessary support and skills [1,2]. Early intervention programmes are usually implemented at the community level in accordance with the economic situation in a given country. In Spain, they have been delivered through early intervention centres (CATs) or child development and early intervention Centres (CDIATs). The activity of CATs and CDIATs are interventions “aimed at the child population aged 0–6 years, the family and the environment, whose objective is to respond as soon as possible to the transitory or permanent needs of children with developmental disorders or at risk of suffering them. These interventions, which must consider the child as a whole, must be planned by a team of professionals with an interdisciplinary or transdisciplinary orientation.” [3] (pp. 12). By definition, early intervention programmes in CATs and CDIATs involve various professionals from different knowledge areas (psychologists, educators, physiotherapists, speech therapists, and occupational therapists, among others) who must coordinate their actions under a holistic intervention model, planning actions systematically and sequentially, and individualising the intervention, which should be based on scientific evidence.

More specifically, work on therapeutic intervention is extremely important in early ages to improve development. Early care therapeutic intervention programs—which can be applied by a range of healthcare professionals, as noted above—are an important reference in predicting the development or progression of a condition. Traditionally, these types of intervention programs have been applied from case interventions [4]. However, as will be noted below, advances in computing and technology can help them be more effective, as long as data from the different healthcare professionals are recorded and shared in databases.

This will involve handling a large volume of information and requires appropriate technical resources. The development of information and communication technologies (ICTs) has positively influenced the control of processes, records, and developments in early intervention programmes by using integrated information systems [5,6]. Databases in which assessments, activities, and outcomes are recorded will enable early childhood practitioners to more accurately analyse the implementation of early intervention programmes. This will help to improve knowledge and future therapeutic interventions. Specifically, intervention records in the form of “medical records” have deep historical roots, although it was not until the middle of the 20th century that the foundations of what are now understood as automated records were laid [7]. The appearance and development of ICTs has led to the emergence of the electronic health record [8,9,10] and defined the functionality of medical record databases. Currently, there is a great effort to develop models that incorporate artificial intelligence (AI) as an aid to decision-making in the diagnostic setting [11], including in early education programmes [12]. The use and development of AI is shaping technological development and revolutionising areas of intervention, particularly early intervention [13].

AI is the science that builds on engineering and computational knowledge by mimicking intelligent human behaviour and applying computer-implemented algorithms. AI includes supervised machine learning (ML) techniques—where researchers train the machine to obtain patterns based on data—and deep learning (DL), where the machine has greater autonomy in learning and reasoning prior to drawing conclusions. In ML and DL, algorithms are applied to a large volume of data in order to make predictions and reveal relationships in the data. These algorithms are capable of iterative learning independent of human involvement [14]. Such computational tools have great potential in health sciences, along with challenges and risks. The benefits include improved diagnosis and intervention for patients or service users. For example, one advantage of DL techniques over traditional methods of data analysis is that the traditional model is based on a series of independent or predictor variables for the dependent variable and DL models can infer relationships within large datasets to establish the most effective predictions [15]. Unsupervised ML techniques (clustering) are used to find the possible effects of a treatment for groups, or profiles, of people to whom a diagnosis or treatment can be assigned [16]. They allow the identification of different types of treatment response patterns in different types of patients (with higher or lower risk). How well these decisions fit depends largely on the amount of data; the larger the volume, the greater the goodness of fit [14].

Furthermore, the framework of digitalisation includes using the internet of things (IoT). This resource allows the transmission and interconnection of data in the cloud and subsequent processing by applying ML or DL techniques. This functionality may help improve diagnostic and treatment decision-making [17,18].

In summary, AI techniques and the IoT are a promising development in health sciences. The most commonly used algorithms are support vector machines (SVMs), convolutional neural networks (CNNs), artificial neural networks (ANNs), random forests, decision trees, and the semantic Web [19]. One systematic review found that the most widely used algorithms for pathology diagnosis were random forests, SVMs, and CNNs [20], while the semantic Web, ontology mining, and topic modelling algorithms are used in clinical or biomedical text mining. On the other hand, ANNs and logistic regression are used to predict variables for both diagnosis and intervention. Finally, CNNs and SVMs are used for observation and classification. A description of the most significant supervised and unsupervised ML techniques and their functionality in the context of health sciences is presented in Table 1.

In summary, using AI together with the IoT is called smart healthcare [24]. The functionality offered by smart healthcare includes prevention and intervention planning for different pathologies [25]. The end result is precision intervention. For example, this type of intervention is being applied very successfully in oncological treatment [26] and in paediatric sepsis [27].

In addition, AI and the IoT have recently been used in psychology [26]. These techniques are being used in the diagnosis of and intervention in mental health problems [28] and in the treatment of degenerative diseases, such as Alzheimer’s disease [29,30]. They are also being used for behavioural prediction in certain neurodegenerative conditions [31,32] and in suicidal risk behaviours [33].

Finally, another of AI’s challenges is how to explain its results—its explainability—and data visualisation techniques are advisable for this. Visualisation of results is essential for health science professionals to be able to smoothly apply these techniques in natural contexts. Furthermore, practitioners’ assessments of explainability will help to identify necessary improvements, which can then be implemented by computer and IT experts [34].

### Application of AI and IoT in Early Intervention in CATs and CDIATs

The key to using AI successfully is to have a large volume of data [35]. All patient services generate large amounts of data, including the early intervention service CATs and CDIATs. However, records are often paper-based rather than being brought together in databases, which is the first step in advancing the application of AI in this area. Just as medical services once promoted the use of databases controlled by medical professionals, early childhood services should promote the same thing. To date, few studies in the field of CATs and CDIATs specifically referring to therapeutic intervention have addressed the applicability of IA and IoT to routine assessment and intervention work in natural contexts. A systematic review [12] analysed the use of AI in educational work with young children, finding very few studies examining this use. Most of those studies focused on behavioural video studies of children’s interactions with robots. However, no studies have been found that use AI and the IoT in early care for children in a similar way to so-called precision medicine—using AI and IoT techniques to identify biomarkers in certain conditions, and on the basis of that, to discriminate between types within the same condition, e.g., cancer [36], or analyse genetic patterns [37] before specific therapies are implemented for each type of condition and their effectiveness is assessed, e.g., absorption mechanisms of drug components [38]. The advantages of this methodology lie in the precision of both diagnosis and intervention.

The same method could also be applied to early intervention contexts for children with different impairments. The first step would require the collection of structured information on developmental progression in different contexts (cognitive, psychomotor, language, socialisation, autonomy and its functioning in family, social, school contexts, etc.). To achieve this, IoT-based tools would have to be developed to enable the unified recording of data related to the development of patients with various impairments. Such tools should include AI techniques to return a personalised development profile. Then, based on this profile, the most appropriate intervention programme would be proposed for each case. Subsequently, programme effectiveness would be monitored [39]. However, there are obstacles, the most notable of which centre on the compilation of extensive databases that would allow the production of reliable indicators for prediction, classification, and pattern analysis in different types of groupings (clusters).

Therefore, this study focuses on developing a working protocol in the field of early care based on the use of IoT and ML techniques to study the advantages and challenges of this methodology in this area. In short, the study objectives are about examining the functionality and application of ML techniques in early care therapeutic intervention. This is specified in the following three objectives:

1. To analyse the functionality of using supervised machine learning techniques for prediction in the group amenable to early care;
2. To analyse the functionality of using supervised machine learning techniques for classification in the group amenable to early care;
3. To analyse the functionality of using unsupervised machine learning clustering techniques in the group amenable to early care.

## 2. Materials and Methods

### 2.1. Design

In accordance with the classification from Campbell and Stanley [40], the study used a descriptive–correlational design.

### 2.2. Participants

We worked with a total sample of 113 service users, 43 of whom were female and 70 of whom were male. The sample was split into two groups. Group 1 comprised 49 patients at a care centre for people with motor impairments (18 female (M_age_ = 150 months; SD_age_ = 66.02); 31 male (M_age_ = 149.03; SD_age_ = 64.10)). Group 2 comprised 64 subjects (25 female (M_age_ = 30.36; SD_age_ = 32.23) and 39 male (M_age_ = 32.82; SD_age_ = 32.19)). Group 2 included 14 patients with motor impairments, 7 with psychomotor delay, 14 with maturational delay, 8 with impairments in different syndromes, 15 with impairments in communication and language development, 5 with autism spectrum impairments, and 1 with visual impairment. All patients in the two groups had functional developmental abilities in the 0–6 years age range, regardless of their chronological age. The sample was chosen by convenience. The two groups were not random; they were based on the possibility of working in two different forms of therapeutic intervention. Group 1 received therapeutic intervention at a care centre for people with motor impairments, whereas Group 2 received interventions as outpatients at home or at health centres. This second group also included other conditions described above, in addition to motor impairments.

### 2.3. Instruments

The eEarlyCare Therapeutic Program web application (eEarlyCare-TP) [39] contains an observation scale for the development of functional skills for individuals aged 0–6 years (FSMS). Specifically, it measures 114 skills grouped into 11 functional areas and 27 sub-areas (see Table S1) rated on a Likert-type scale from 1 to 5 (1 = not at all and 5 = All). The FSMS was produced by consulting the Brunet–Lézine Scale [41], the Batelle inventory [42] and the PEDI scale [43]. eEarlyCare-TP also includes therapeutic intervention programmes for the development of functional skills [39]. The development of these programmes was inspired by the structure of the Portage Guide [44].

Similarly, eEarlyCare-TP applies a learning analytics system that can produce a development profile for each patient in one, two, or three measurements. The tool allows development profiles to be compared between different patients [39]. An example is shown in Figure S1. The tool also creates customised programmes according to each development profile, an example of which is shown in Figure S2.

The tool has a Cronbach’s alpha reliability index of α = 0.88 for the full scale and a range of α = 0.85–α = 0.91 if the item is removed, along with a McDonald’s omega reliability index of ω = 0.96 and a range of ω = 0.94–ω = 0.96 if the item is removed. In this study, the reliability indicators were α = 0.98 for the full scale and an interval of α = 0.94–α = 0.95 if the item is removed, and a McDonald’s omega reliability index of ω = 0.97 and an interval of ω = 0.96–ω = 0.97 if the item is removed. These instruments were produced in the framework of funding from the European Regional Development Fund (FEDER), more specifically from three proof-of-concept projects, VI, VII, and VIII, financed by the government of Castille and León in Spain (more information in [39]).

### 2.4. Procedure

**Figure 1:**
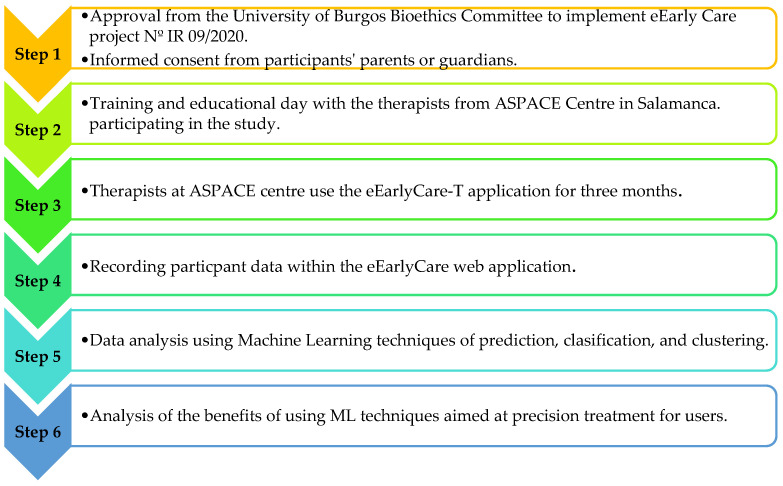
Steps in the procedure applied in this study.

Firstly, the study was approved by the Bioethics Committee of the University of Burgos, No. IR 09/2020. This work was carried out at the ASPACE SALAMANCA Centre; ASPACE is an association of parents, tutors, and guardians who, together with highly qualified professional staff with extensive experience, seek to achieve the same daily objective of offering people with cerebral palsy a space where they can grow with opportunities. More information can be found at https://aspacesalamanca.org/ (accessed on 20 March 2024). ASPACE SALAMANCA also offers an early intervention programme for children aged 0–6 years, the aim of which is to respond as quickly as possible to the temporary or permanent needs of children with or at risk of suffering from diagnostic disorders. The work is focused on children and their families. More information can be found at https://aspacesalamanca.org/centros-y-servicios/atencion-temprana/, accessed on 20 March 2024)). In this centre, the informed consent of the families or legal guardians of the children participating in the study was obtained. Therapists then used the eEarlyCare web-based application for three months. Data were recorded in the application and then analysed using supervised (prediction and classification) and unsupervised (clustering) machine learning techniques. Finally, the advantages and challenges of using these techniques in the field of therapeutic intervention at an early age were studied. Figure 1 shows a schematic of the above procedure.

### 2.5. Data Analysis

To address objective 1, linear regression analysis was applied. To address objective 2, the supervised learning algorithm classification decision tree was applied. To address objective 3, the *k*-means unsupervised learning algorithm was used. Beforehand, the elbow method was used to check the number of clusters. Also, a cross-tabulation table was applied to check the relationship between the assignment node or cluster of membership and the research group (Group 1 vs. Group 2). These analyses were carried out using SPSS v.28 [45]. In addition, Cronbach’s alpha and omega reliability indicators for this study were examined using SPSS v.28 [45]. Orange data mining software was used to obtain the visualisation images [46]. Table 2 presents an outline of the analyses conducted with respect to the study objectives.

## 3. Results

### 3.1. Analysing the Functionality of Using Supervised Machine Learning Techniques for Prediction in the Group Amenable to Early Care

To test objective 1, a linear regression analysis examined the predictive effect of the age variable on the results of patients’ functional abilities. A distinction was made between patients in Group 1 (care for people with motor impairment) and Group 2 (care for patients at an early age in the early detection programme). Group 1 demonstrated R^2^ = 0.85. This indicates that the chronological age of the patients predicts 85% of the results in the functional skills scale. Group 2 demonstrated R^2^ = 0.65. This indicates that chronological age in these patients predicts 65% of the results in the functional skills scale. These results suggest that working at an early age would explain 20% of the development results in these types of skills. In addition, as the patients in Group 2 had different impairments, the effect of the variable ‘type of impairment’ on the development of functional skills was examined, giving R^2^ = 0.64, indicating that this variable explains 64% of functional development. The type of impairment and chronological age are two key factors to consider when preparing therapeutic intervention programmes. This is in line with the importance of carrying out early stimulation programmes as early as possible [4].

### 3.2. Analysing the Functionality of Using Supervised Machine Learning Techniques for Classification in the Group Amenable to Early Care

**Figure 2:**
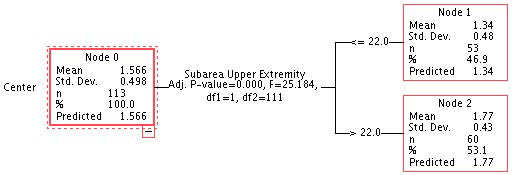
Decision tree applied to the results in FSMS in the two groups.

To examine objective 2, the CHAID decision tree classification algorithm was applied with respect to the dependent variable type of intervention centre (a specific early intervention centre vs. a non-specific centre). The decision tree algorithm facilitates the hierarchical organization of questions for guiding the process of assigning classes. The process begins in the root node, where values of one of the attributes of the instance to classify is offered and proceeds along various branches to nodes with new branches, where a class is assigned to the instance [22,23]. The variables with the greatest weight in the classification were the scores in the upper extremity sub-area, which differentiated two groups, scores above 22 (n = 53) and below 22 (n = 60), in FSMS (the maximum score in this sub-area was 25 points). In the first group, the mean score in the upper extremity sub-area was 1.34 out of 5, while in the second group it was 1.77, both with an SD of less than 1 (see Figure 2).

Next, a cross-tabulation table was created to examine the distribution of Group 1 and Group 2 patients in the nodes found in the decision tree. As Table 3 shows, 31% of the total patients assigned to node 1 belonged to Group 1 (representing 71.42% of the total patients in Group 1 (scores below 22 in the upper extremity sub-area)) and 40% of the total patients belonged to Group 2 (representing 71.88% of the total patients in Group 2 (scores above 22 in the upper extremity sub-area)).

Based on this result, the CHAID decision tree algorithm for the dependent variable “type of diagnosis” in relation to scores in the FSMS areas and sub-areas was applied to the Group 2 sample “specific early care centre” (it was not applied to Group 1, as all members of that group were included in the motor impairment category). In this case, no rankings were found for any independent variable with respect to the dependent variable diagnosis type. In both cases, cross-validation with 10 sample folds was applied.

### 3.3. Analysing the Functionality of Using Unsupervised Machine Learning Clustering Techniques in the Group Amenable to Early Care

For the third objective, the *k*-means clustering algorithm was applied. Before that, the elbow method was used to determine the number of clusters, and the optimal k was found to be 2 (see Figure S3).

In Group 1, two clusters and significant differences were found in all functional areas except for adaptive behaviour. In this group, the highest scores in the FSMS areas were found in cluster 2 (see Table 4).

A visual analysis of the clusters was also performed; this can help the practitioner to visualise each patient’s functional development pattern (see Figure S4).

Next, a cross-tabulation table was made between the age categorisation and the cluster number assigned to each patient in Group 1 (see Table 5). In this group, the patients had chronological ages that reached 288 months, as Table 5 shows, and cluster 2 (in which the highest values for development of the functional areas was detected) included patients from all four age groups.

**Figure 3:**
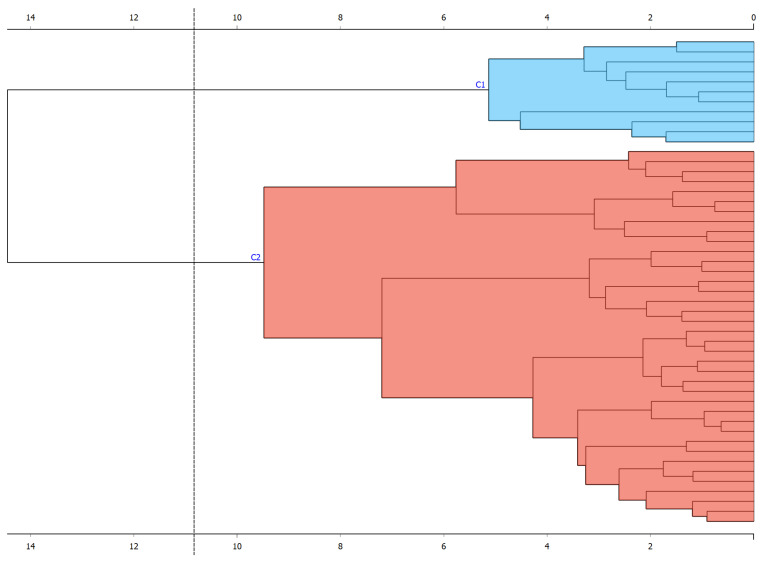
Hierarchical clustering in Group 1.

In Group 1, the participants had the same diagnosis (motor impairment). The hierarchical clustering was applied with the Ward method (considered the most suitable for quantitative data). Two hierarchical clusters were found that included each participant (see Figure 3). In addition, principal component analysis (PCA) was performed for this group; two components were selected that explained 60% of the variance (see Figure S5).

For Group 2, the elbow method was applied beforehand to determine the optimal number of clusters, indicating *k* = 2 (see Figure S6). Then, *k*-means was applied with tow clusters. Table 6 shows the scores in each cluster for the FSMS areas of functional development; the highest scores were found in cluster 1. Significant differences were also found in all areas of development except interactive and symbolic play, adaptive behaviour, and attention.

A visual analysis of the clusters was also performed; this can help the practitioner to visualise each patient’s functional development pattern (see Supplementary Figure S7).

The relationship between diagnosis type and assignment cluster was then analysed by cross-tabulating the diagnosis type and assignment cluster (see Table 7).

Participants with diagnoses 5 (impairment in communication and language development), 6 (autism spectrum), and 7 (visual impairment) were only found in cluster 1, which is where the highest scores in the developmental areas were recorded.

**Figure 4:**
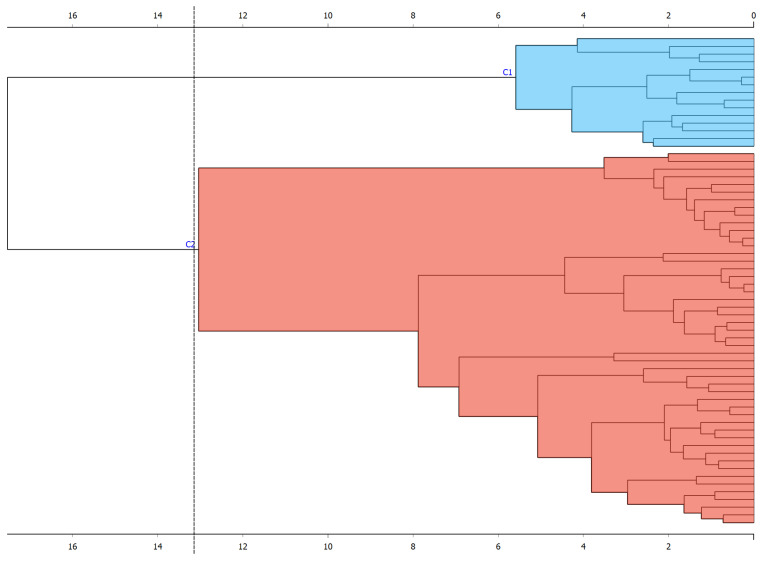
Hierarchical clustering in Group 2.

In addition, the hierarchical clustering algorithm was applied with the Ward method. Two hierarchical clusters were found that included each participant (see Figure 4). In addition, PCA was performed for this group; two components were selected that explained 64% of the variance (see Figure S8).

## 4. Discussion

The use of supervised ML prediction techniques allows us to determine the prediction percentage of certain variables in the study groups [15]. For example, in this study, we worked with two groups of patients with different characteristics. Although both groups had a developmental age in functional skills in the 0–6 year range, a priori, they had different characteristics. Group 1 was made up of people with motor impairments who were chronologically over six years old and who were being treated in a care centre for people with motor impairments. In contrast, Group 2 was made up of patients chronologically aged between 0 and 6 years old who participated in an outpatient early care programme [4]. The variables affecting functional development in the two groups were different. In Group 1, the dominant pathology was the previously established motor impairment in patients over 6 years of age. The chronological age variable in this group explained 85% of the variance in the development of functional skills. However, in Group 2 (where the chronological age of the patients was in the range of 0–6 years old), chronological age explained 65% of the variance in the results on the development of functional skills, and the type of impairment (diagnosis) explained 64%. These results underscore the importance of early intervention for promoting the development of functional skills, since there was a 20% difference between the two groups in the weight of the variable ‘chronological age’ on functional development [4].

In relation to objective 2, the ML classification techniques made it possible to detect which functional development variables were key. In this case, the variable with the greatest weight in the development of functional skills was the sub-area of development in the upper extremities. A greater problem in this development was detected in Group 1 patients than in those in Group 2. There are a variety of potential explanations, although it is worth highlighting the importance of early stimulation in facilitating overall functional development. It is also important to differentiate the patient’s type and degree of impairment with respect to functional development and to propose individualised programmes that promote work on the most affected areas. These data are important in order to propose therapeutic intervention programmes that are more precise and a better fit to each patient’s pathology and impairment [25].

Once we had established the difference in functional development between patients in Group 1 and Group 2, cluster analysis was applied separately to each group using the *k*-means algorithm. This algorithm provides information on the clustering of patients within each reference group without applying a prior assignment variable—in contrast to supervised ML techniques. This functionality has the advantage of being able to analyse, specifically in this case, clustering in the development of functional skills within each group (Group 1 and Group 2). The specific detection of each patient in each cluster makes it possible to determine each patient’s position with respect to functional development in the different areas analysed. This information will guide the development of similar therapeutic intervention programmes for patients with similar functional characteristics. On the one hand, this will facilitate more precise intervention, and on the other, it will allow early intervention centres to make the most of their resources. In addition, applying data visualisation techniques to clustering algorithms will make it easier for early care professionals to rapidly analyse results and better understand them [34]. This study has shown that the interpretation of functional development in different types of patients can be read differently depending on other variables, such as chronological age and type of impairment [18,39]. In addition, applying ML techniques made it possible to study patients’ characteristics (age; type of impairment) and the significance of those characteristics in the results found. Using ML techniques also guided individualised intervention for each patient [39]. In summary, early therapeutic intervention through individualized programs is the key to each user successfully recovering their abilities as far as possible. This has always been the core of early care [4]. Nonetheless, using ML techniques such as non-supervised clustering can improve the precision of how certain types of programmes are applied for each user with different impairments. This is the current dynamic in what is called “precision medicine”, and one we believe should be transferred to early therapeutic care. However, that would need secure storage networks of anonymized data that could collect patient characteristics (diagnosis, age, age at diagnosis and intervention, type of programme applied, duration, follow-up results, etc.).

### 4.1. Limitations of this Study

This study was only intended as an example of how some smart healthcare resources can be used in the field of early care. We worked with a specific web application, eEarlyCare-TP, which analyses functional skills in developmental ages 0–6 years. However, similar web applications should be developed to analyse other developmental skills, such as cognitive and metacognitive precursors. In addition, we worked with a specific population sample, although it was possible to differentiate between patients participating in a specific early attention programme with chronological ages 0–6 years and patients with a functional development of 0–6 years but chronologically aged over 6. Although this is not a generalisation of the results, it did allow analysis with a sample of n = 113 participants, a ratio that is not the norm in this type of study. In any case, future research should attempt to standardize these instruments in different population samples in order to improve validity and reliability, although we need to recognize the difficulties of working with large volumes of data in these types of populations. Furthermore, ML techniques were applied by extracting the database from the web application and analysing the data with statistical analysis programmes and Python visualisation packages. This means that, as of now, those techniques cannot be applied to work in natural contexts, as result analysis is not yet automated in the web application and programming knowledge is needed for that.

### 4.2. Future Lines of Intervention

The smart healthcare approach [25] applied to early care still has difficulties and challenges to be addressed. Firstly, large databases are needed to more accurately apply ML and DL techniques [36]. Therefore, the recording of data on the development of patients with different impairments at developmental ages 0–6 years should be unified at a national and international level. To achieve this, developmental assessment instruments must be created and implemented in web applications, and health science professionals working with these groups (neonatologists, paediatricians, child psychologists, rehabilitation doctors, stimulators, physiotherapists, speech therapists, occupational therapists, etc.) should be encouraged to use them in both public and private services. Then, supervised and unsupervised ML algorithms and data visualisation techniques can be applied to those data in order to return the resulting information to the professionals in a simple way. Currently, the application and interpretation of ML techniques still requires significant computer expertise. There needs to be an automated implementation of these algorithms in computer applications that are easy for practitioners to use and interpret in daily practice. In addition, having a large volume of data available will allow an examination of what type of treatment is most effective for patients with similar characteristics in terms of the type and degree of impairment—as currently happens for medical impairments in precision therapies [39]. Finally, having large databases would facilitate the application of not only ML but also DL techniques, and this functionality would mean a breakthrough in the use of AI for diagnosis and intervention in the field of early care. The challenge of using ML techniques in early care is also an opportunity. The challenge is being able to achieve shared databases in the same way as other medical and healthcare disciplines have. The opportunity is in applying supervised and unsupervised ML techniques to these databases, which will no doubt demonstrate the effectiveness of therapeutic interventions for various conditions and ages, and various intervention settings.

## 5. Conclusions

In summary, we can conclude that there is a promising future for IoT and ML techniques (both supervised and unsupervised) within what has been called smart healthcare [24] applied to the field of early intervention. Firstly, recording information in web applications makes it easier to record and subsequently extract data. Furthermore, studying that data by applying supervised and unsupervised ML techniques allows professionals to analyse and interpret the data, often visually. This functionality provides the practitioner with a lot of information that cannot be gathered from observation alone or from using simpler data analysis techniques. Along similar lines, detecting patients’ differentiating characteristics and the variables that have more of an impact on the results of an intervention will allow professionals in early intervention programmes to make more precise diagnoses and interventions. In turn, this will foreseeably improve the quality of life of patients and their families and will improve the distribution of personal and material resources in early intervention centres.

## Figures and Tables

**Table 1:** Machine learning techniques and applicability in early care.

Techniques	Algorithms That Apply	Use in Health Sciences	Techniques
Supervised ML Techniques			
Classification			
SVM	Based on Vapnik’s theory [21]. In two linearly separated classes, the best boundary between two classes is searched. In non-linear functions, the kernel trick is applied.		Facilitates visual analysis
Discriminant Analysis	Describe whether there are significant differences in *x* groups and variables. It is a prediction model of a categorical response variable, *x*, from y classifier variables that are usually continuous.	Detection of proxy variables to adjust a diagnosis or best treatment	Facilitates visual analysis
Nearest Neighbour	A non-parametric classification and regression method that estimates the probability density function that an element, *x*, belongs to a class, *C*_i._ It is known as a lazy technique. The most frequently applied distance is Euclidean.	Can help in grouping types of patients within a pathology in relation to the degree of involvement. May also help in pooling the effectiveness of different types of treatment in different types of patients.	Facilitates visual analysis
Decision Tree	An algorithm that detects the influence of a series of variables (independent) on other variables (dependent) in a hierarchical order. It is quick to build, interpretable, and sensitive to small changes. It can construct multiclassifiers [22] and regressors [23].	Can detect the most effective treatment among the possible treatments or the part of a treatment in a hierarchical order of percentage explained with respect to the results of an intervention.	Facilitates a visual analysis that is highly intuitive.
Neural Networks	Computational models that emulate human neural functioning. They include: Multi-layer Perceptron NN. Can solve problems that are not linearly separable. Outputs can be imprecise. Radial-Based Neural Networks. They can construct linear and non-linear approximations.	Used to solve problems of pattern association, image segmentation or data understanding. Time series analysis, image processing, speech recognition, and diagnostics.	Facilitates visual analysis
Prediction			
Linear regression	A model used to approximate the relationship between continuous variables, a dependent variable, other independent variables, and an error variable.	Can help predict the effect of a risk factor on the development of a pathology or the effect of a type of treatment on the symptoms and progression of a pathology. Applied to variables that are measured on an interval or ratio scale.	Facilitates visual analysis
Logistic regression	A similar model to linear regression that predicts the outcome of a categorical variable with respect to predictor variables. It examines binomially distributed data.	Can help predict the effect of a risk factor on the development of a pathology or the effect of a type of treatment on the symptoms and progression of a pathology. Applied to variables that are measured on a dichotomous scale.	Facilitates visual analysis
Unsupervised ML techniques			
Clustering			
*k*-means	Allows the assignment of an element to a cluster without applying a prior clustering variable. The assignment is made with the closest distance to the centre of that cluster. The disadvantage is that the algorithm tends to form groups of similar sizes.	Allows the determination of groupings of patients without a previously defined independent variable with respect to different measurements in different relevant parameters.	It facilitates a visual analysis that is highly intuitive in this case.
*k*-means ++	Solve the *k*-means NP hard problem. To do so, apply a polynomial transformation and then run the cluster centre assignment algorithm.	Allows for a tighter distribution of the grouping of patients without a pre-defined independent variable for different measurements on different relevant parameters.	Facilitates a visual analysis that is highly intuitive in this case.

**Table 2:** Study objectives and data analysis tests.

Study Objectives	Data Analysis Tests
1. To analyse the functionality of using supervised machine learning techniques for prediction.	Supervised machine learning prediction technique: linear regression
2. To analyse the functionality of using supervised machine learning for classification.	Supervised machine learning technique for classification: decision tree (CHAID algorithm) Cross-tabulation table
3. To analyse the functionality of using unsupervised machine learning clustering techniques.	Unsupervised machine learning technique: *k*-means clustering; hierarchical clustering cross-tabulation table principal component analysis (PCA) (elbow method).

**Table 3:** Cross-tabulation table between the type of group (patients in Group 1 vs. patients in Group 2) and the allocation node in the decision tree.

Group		Node		
	1	%	2	%
	n = 53		n = 60	
1	35	30.97	14	12.39
2	18	15.93	46	40.71
Total	53	46.90	60	53.10

Note. Group 1 = patients with functional developmental progress aged 0–6 years from a specific centre for people with motor impairment; Group 2: patients with different impairments (physical, mental, and sensory) at chronological ages 0–6 years who participate in a specific early care programme.

**Table 4:** Clusters and ANOVA in Group 1.

FSMS Areas and Sub-Areas	Cluster 1	Cluster 2	*F*	*p*
	n = 35	n = 14		
Food autonomy	12	29	74.16	<0.001 *
Personal care and hygiene	25	56	64.21	<0.001 *
Dressing and undressing autonomy	18	52	83.52	<0.001 *
Sphincter control	8	24	91.45	<0.001 *
Functional mobility	59	133	58.48	<0.001 *
Communication and language	24	43	21.88	<0.001 *
Task solving in social contexts	9	13	4.52	0.04 *
Interactive and symbolic play	11	26	11.28	<0.001 *
Routines in daily life	3	7	10.69	<0.001 *
Adaptive behaviour	11	13	0.40	0.53
Attention	4	5	0.47	0.50 *

Note. FSMS = scale for the development of functional skills for ages 0–6 years; *p* < 0.05 *.

**Table 5:** Cross-tabulation between age categorisation and assigned clusters in Group 1.

Age Group	Cluster	
	1 n = 35	2 n = 14
1	9	1
2	14	3
3	6	7
4	6	3

Note. 1 = 0–72 months; 2 = 73–144 months; 3 = 145–216 months; 4 = 217–288 months.

**Table 6:** Clusters and ANOVA in Group 2.

FSMS Areas and Sub-Areas	Cluster 1	Cluster 2	*F*	*p*
	n = 34	n = 30		
Food autonomy	31	13	109.84	<0.001 *
Personal care and hygiene	63	27	141.30	<0.001 *
Dressing and undressing autonomy	52	19	145.20	<0.001 *
Sphincter control	19	7	33.89	<0.001 *
Functional mobility	140	63	135.85	<0.001 *
Communication and language	39	20	58.32	<0.001 *
Task solving in social contexts	10	6	5.23	0.026 *
Interactive and symbolic play	27	20	2.93	0.093
Routines in daily life	7	4	7.03	0.010 *
Adaptive behaviour	14	11	1.65	0.204
Attention	5	5	0.008	0.929

Note. FSMS = scale for the development of functional skills for those aged 0–6 years; *p* < 0.05 *.

**Table 7:** Cross-tabulation between age categorisation and assigned clusters in Group 2.

Diagnoses Type	Cluster	
	1 n = 34	2 n = 30
1	4	10
2	3	6
3	4	8
4	2	6
5	15	0
6	5	0
7	1	0

Note. 1 = motor impairment; 2 = psychomotor retardation; 3 = maturational delay; 4 = various syndromes; 5 = impairment in communication and language development; 6 = autism spectrum; 7 = visual impairment.

## Data Availability

The data of this pilot study may be requested upon written request from the university or institution that endorses that the data will be used for scientific purposes and after signing a data protection agreement with the data protection officer of the University of Burgos. The data are not publicly available due to the University of Burgos’s data protection policy.

## Supplementary Materials

The following supporting information can be downloaded at https://www.mdpi.com/article/10.3390/children11040381/s1: Table S1: Functional areas and sub-areas in FSMS; Figure S1: Example of profiling with the eEarlyCare-TP web application; Figure S2: Example of profiling with the eEarlyCare-TP web application; Figure S3: Elbow method in ***k***-means in Group 1; Figure S4: *k*-means patterns in Group 1; Figure S5: Principal component analysis in Group 1; Figure S6: Elbow method in *k*-means in Group 2; Figure S7: *k*-means patterns in Group 2; Figure S8: Principal component analysis in Group 2.

## References

[1] Blackman J.A. (2002). Early Intervention: A Global Perspective. Infants Young Child..

[2] Peterander F., Speck O., Pithon G., Terrisse B. (1999). Les Tendances Actuelles de L’intervention Précoce en Europe.

[3] Federación Estatal de Asociaciones de Profesionales de Atención Temprana (GAT) (2005). Libro Blanco Atencion Temprana.

[4] Gómez Artiga A., Viguer Seguí P., Cantero López M.J. (2005). Intervención Temprana: Desarrollo Óptimo de 0 a 6 Años [Early Intervention: Optimal Development from 0 to 6 Years of Age].

[5] Anderson R.G. (1987). Development of Business Information Systems.

[6] Anderson R.G. (1992). Information and Knowledge-Based Systems: An Introduction.

[7] Siegler E.L. (2010). The evolving medical record. Ann. Intern. Med..

[8] Dick R.S., Steen E.B., Detmer D.E. (1997). The Computer-Based Patient Record: An Essential Technology for Bealth Care.

[9] Aspden P., Corrigan J.M., Wolcott J., Erickson S.M. (2004). Committee on Data Standards for Patient Safety: Achieving a New Standard for Care.

[10] Schiff G.D., Bates D.W. (2010). Can electronic clinical documentation help prevent diagnostic errors?. N. Engl. J. Med..

[11] Sierra I., Díaz-Díaz N., Barranco C., Carrasco-Villalón R. (2022). Artificial Intelligence-Assisted Diagnosis for Early Intervention Patients. Appl. Sci..

[12] Su J., Yang W. (2022). Artificial intelligence in early childhood education: A scoping review. Comput. Educ. Artif. Intell..

[13] Chan S., Ding Z., Lee T.-l., Sze S.L., Yang N.S. (2022). Temporal processing deficit in children with attention-deficit/hyperactivity disorder: An online assessment. Digit. Health.

[14] Makhni S., Chin M.H., Fahrenbach J., Rojas J.C. (2022). Equity Challenges for Artificial Intelligence Algorithms in Health Care. Chest.

[15] Eloranta S., Boman M. (2022). Predictive models for clinical decision making: Deep dives in practical machine learning. J. Intern. Med..

[16] Alcantud-Marín F., Pérez-Bou J., Alonso-Esteban Y. (2019). Diagnostic validity of the Merrill Palmer-R Development Scale used in the evaluation of access to Child Development Centers and Early Care. Siglo Cero.

[17] Al-rawashdeh M., Keikhosrokiani P., Belaton B., Alawida M., Zwiri A. (2022). IoT Adoption and Application for Smart Healthcare: A Systematic Review. Sensors.

[18] Sáiz-Manzanares M.C., García Bringas P., García H.P., de Pisón F.J.M., Álvarez F.M., Lora A.T., Herrero Á., Rolle J.L.C., Quintián H., Corchado E. (2023). Using Machine Learning Techniques in eEarlyCare Precision Diagnosis and Intervention in 0–6 years Old. Proceedings of the International Joint Conference 16th International Conference on Computational Intelligence in Security for Information Systems (CISIS 2023) 14th International Conference on EUropean Transnational Education (ICEUTE 2023).

[19] Alotaibi G., Awawdeh M., Farook F.F., Aljohani M., Aldhafiri R.M., Aldhoayan M. (2022). Artificial intelligence (AI) diagnostic tools: Utilizing a convolutional neural network (CNN) to assess periodontal bone level radiographically—A retrospective study. BMC Oral Health.

[20] Chen X., Cheng G., Wang F.L., Tao X., Xie H., Xu L. (2022). Machine and cognitive intelligence for human health: Systematic review. Brain Inform..

[21] Ryabko D. Algorithmic Learning Theory. Proceedings of the 27th International Conference, ALT 2016.

[22] Maudes J., Rodríguez J.J., García-Osorio C., García-Pedrajas N. (2012). Random feature weights for decision tree ensemble construction. Inf. Fusion.

[23] Arnaiz-González Á., Díez-Pastor J.F., Rodríguez J.J., García-Osorio C. (2016). Instance selection for regression: Adapting DROP. Neurocomputing.

[24] Manickam P., Mariappan S.A., Murugesan S.M., Hansda S., Kaushik A., Shinde R., Thipperudraswamy S.P. (2022). Artificial Intelligence (AI) and Internet of Medical Things (IoMT) Assisted Biomedical Systems for Intelligent Healthcare. Biosensors.

[25] Parida P.K., Dora L., Swain M., Agrawal S., Panda R. (2022). Data science methodologies in smart healthcare: A review. Health Technol..

[26] Lin B., Wu S. (2022). Digital Transformation in Personalized Medicine with Artificial Intelligence and the Internet of Medical Things. Omics J. Integr. Biol..

[27] Chen X., Zhang R., Tang X., Qian J. (2022). Prediction of Pediatric Sepsis Using a Deep Encoding Network with Cross Features. J. Shanghai Jiaotong Univ. (Sci.).

[28] Jones M.N. (2017). Big Data in Cognitive Science.

[29] Zhou S., Zhao J., Zhang L. (2022). Application of Artificial Intelligence on Psychological Interventions and Diagnosis: An Overview. Front. Psychiatry.

[30] Liu Q., Vaci N., Koychev I., Kormilitzin A., Li Z., Cipriani A. (2022). Personalised treatment for cognitive impairment in dementia: Development and validation of an artificial intelligence model. BMC Med..

[31] Alty J., Bai Q., Li R., Lawler K., St George R.J., Hill E., Bindoff A., Garg S., Wang X., Huang G. (2022). The TAS Test project: A prospective longitudinal validation of new online motor-cognitive tests to detect preclinical Alzheimer’s disease and estimate 5-year risks of cognitive decline and dementia. BMC Neurol..

[32] Nichol B.A.B., Hurlbert A.C., Read J.C.A. (2022). Predicting attitudes towards screening for neurodegenerative diseases using OCT and artificial intelligence: Findings from a literature review. J. Public Health Res..

[33] Aldhyani T.H.H., Alsubari S.N., Alshebami A.S., Alkahtani H., Ahmed Z.A.T. (2022). Detecting and Analyzing Suicidal Ideation on Social Media Using Deep Learning and Machine Learning Models. Int. J. Environ. Res. Public Health.

[34] Dey S., Chakraborty P., Kwon B.C., Dhurandhar A., Ghalwash M., Saiz F.J.S., Ng K., Sow D., Varshney K.R., Meyer P. (2022). Human-centered explainability for life sciences, healthcare, and medical informatics. Patterns.

[35] Spathis D., Perez-Pozuelo I., Marques-Fernandez L., Mascolo C. (2022). Breaking away from labels: The promise of self-supervised machine learning in intelligent health. Patterns.

[36] Dawoud A., Ihab Zakaria Z., Hisham Rashwan H., Braoudaki M., Youness R.A. (2023). Circular RNAs: New layer of complexity evading breast cancer heterogeneity. Non-Coding RNA Res..

[37] Zhu K., Wu J., Li G., Chen X., Luo M.Y. (2023). A model and cooperative co-evolution algorithm for identifying driver pathways based on the integrated data and PPI network. Expert Syst. Appl..

[38] Li J., Zhang Y., Liu S., Li W., Sun Y., Cao H., Wang S., Meng J. (2023). A network pharmacology integrated pharmacokinetics strategy to investigate the pharmacological mechanism of absorbed components from crude and processed Zingiberis Rhizoma on deficiency-cold and hemorrhagic syndrome. J. Ethnopharmacol..

[39] Sáiz-Manzanares M.C., Marticorena-Sánchez R., Arnaiz-González Á. (2022). Improvements for Therapeutic Intervention from the Use of Web Applications and Machine Learning Techniques in Different Affectations in Children Aged 0–6 Years. Int. J. Environ. Res. Public Health.

[40] Campbell D.F., Stanley J. (2005). Diseños Experimentales y Cuasiexperimentales en la Investigación Social.

[41] Josse D. (1997). Scale of Psychomotor Development of Early Childhood Brunet Lézine-Revised.

[42] Newborg J. (2005). Battelle Developmental Inventory Examiner’s Manual.

[43] Haley S.M., Coster W.J., Ludlow L.H., Haltiwanger J.T., Andrellos P. (2012). The Pediatric Evaluation of Disability Inventory (PEDI).

[44] Bluma M.S., Shearer M.S., Frohman A.H., Hilliard J. (1978). Portage Guide to Early Education.

[45] IBM Corp (2023). SPSS Statistical Package for the Social Sciences (SPSS), Version 28.

[46] Demšar J., Curk T., Erjavec A., Gorup Č., Hočevar T., Milutinovič M., Možina M., Polajnar M., Toplak M., Starič A. (2013). Orange: Data Mining Toolbox in Python. J. Mach. Learn. Res..

