# Supplementary material for: Use of Digitalisation and Machine Learning Techniques in Therapeutic Intervention at Early Ages: Supervised and Unsupervised Analysis
Source: Children (Basel). 2024 Mar 22;11(4):381. doi: 10.3390/children11040381 (PMC11048911; doi:10.3390/children11040381)
Supplement: Supplementary file 1 [file children-11-00381-s001.zip › children-2870151-supplementary.pdf]

children

MDPI

Supplementary Materials

Table S1. Functional areas and sub-areas in FSMS.

Functional areas and sub-areas	No. of items Maximum score	No. of items Maximum score
**Area food autonomy**	**7**	**35**
Subarea food texture	1	5
Subarea use of utensils	6	30
**Area personal care and hygiene**	**16**	**80**
Subarea dental hygiene	4	20
Subarea hairstyle	3	15
Subarea nasal care	2	10
Subarea handwashing	4	20
Subarea face and body wash	3	15
**Area independently dresses and undresses**	**15**	**75**
Subarea dressing and undressing (waist upwards)	6	30
Subarea dressing and undressing (waist downwards)	9	45
**Area sphincter control**	**7**	**35**
**Area functional mobility**	**30**	**150**
Subarea upper extremity	5	25
Subarea transfers in WC	3	15
Subarea transfers on a chair	2	10
Subarea mobility and bed transfers	3	15
Subarea mobility in the bathtub	2	10
Subarea mobility indoors	5	25
Subarea transport of objects	4	20
Subarea outdoor mobility	6	30
**Area communication and language**	**10**	**50**
Subarea word comprehension	3	15
Subarea understanding phrases	3	15
Subarea functional use of communication	4	20
**Area resolution of tasks in social contexts**	**5**	**25**
Subarea solving problems	2	10
Subarea information about yourself	3	15
**Area interactive and symbolic play**	**10**	**50**
Subarea interactive play	3	15
Subarea interaction with the peer group	2	10
Subarea play with objects	5	25

**Area daily life routines**	**3**	**15**
Subarea guidance in time	1	5
Subarea homework	2	10
**Area adaptive behavior**	**9**	**45**
Subarea self-harm behavior	2	10
Subarea heteroagressiveness (harming others)	3	15
Subarea destruction of objects	1	5
Subarea disruptive behaviour (cry, shout, laugh without reason	1	5
Subarea stereotypes	2	10
**Area attention**	**2**	**10**

Note. FSMS = Scale for the development of functional skills for ages 0-6 years.

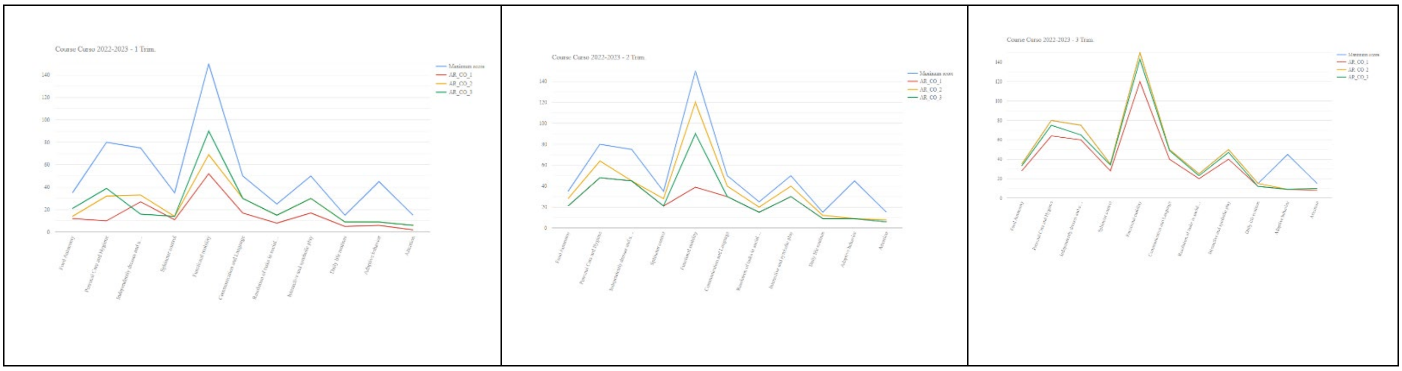

Figure S1. Example of profiling with the eEarlyCare-TP web application.

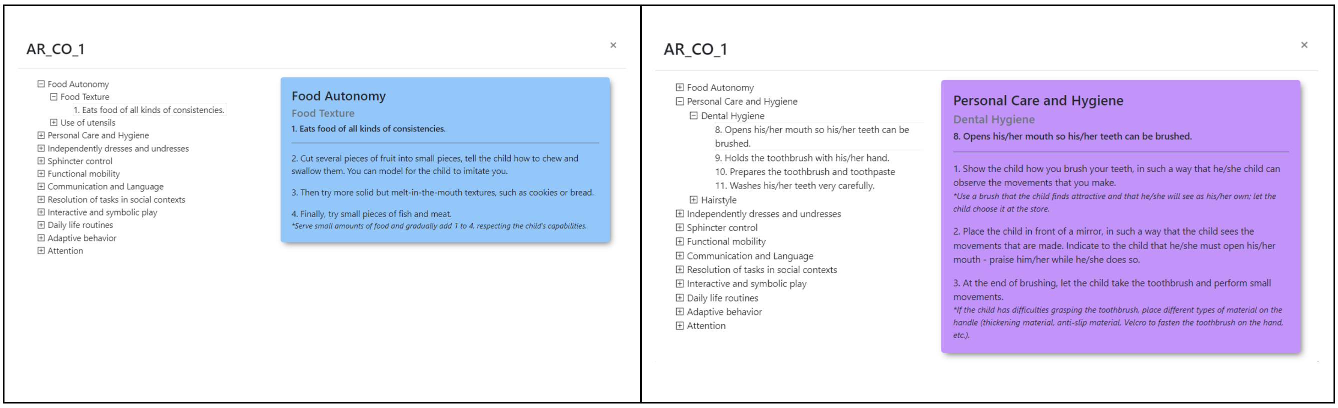

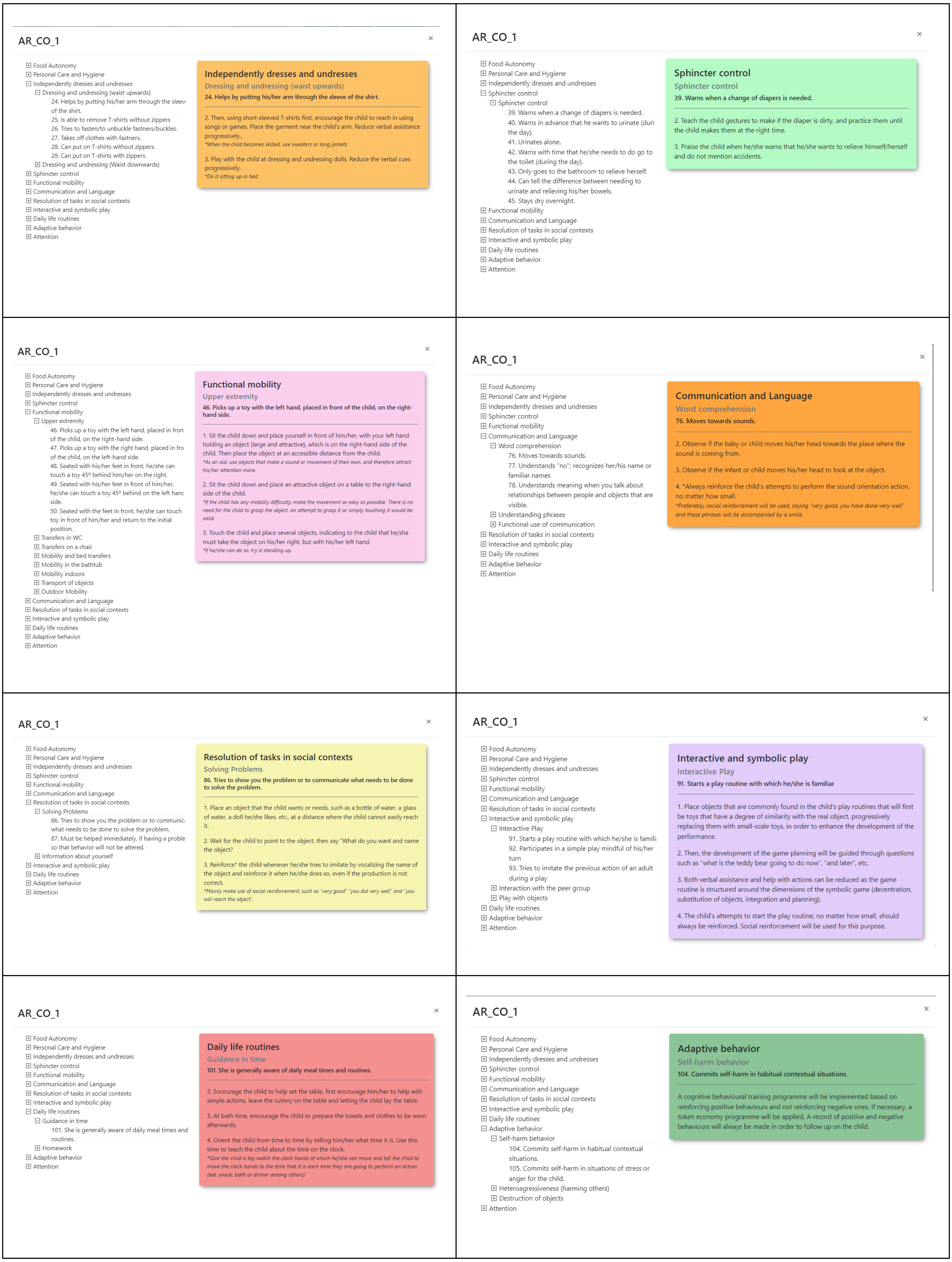
AR_CO_1
Food Autonomy
Personal Care and Hygiene
Independently dresses and undresses
Dressing and undressing (waist upwards)
24. Helps by putting his/her arm through the sleeve of the shirt.
25. Is able to remove T-shirts without zippers
26. Tries to fasten/to unbuckle fastners/buckles.
27. Takes off clothes with fastners.
28. Can put on T-shirts without zippers.
29. Can put on T-shirts with zippers.
Dressing and undressing (Waist downwards)
Sphincter control
Functional mobility
Communication and Language
Resolution of tasks in social contexts
Interactive and symbolic play
Daily life routines
Adaptive behavior
Attention
Independently dresses and undresses
Dressing and undressing (waist upwards)
24. Helps by putting his/her arm through the sleeve of the shirt.
2. Then, using short-sleeved T-shirts first, encourage the child to reach in using songs or games. Place the garment near the child's arm. Reduce verbal assistance progressively..
*When the child becomes skilled, use sweaters or long jackets
3. Play with the child at dressing and undressing dolls. Reduce the verbal cues progressively.
*Do it sitting up in bed.
AR_CO_1
Sphincter control
39. Warns when a change of diapers is needed.
40. Warns in advance that he wants to urinate (during the day).
41. Urinates alone.
42. Warns with time that he/she needs to do go to the toilet (during the day).
43. Only goes to the bathroom to relieve herself.
44. Can tell the difference between needing to urinate and relieving his/her bowels.
45. Stays dry overnight.
Sphincter control
Sphincter control
39. Warns when a change of diapers is needed.
2. Teach the child gestures to make if the diaper is dirty, and practice them until the child makes them at the right time.
3. Praise the child when he/she warns that he/she wants to relieve himself/herself and do not mention accidents.
AR_CO_1
Functional mobility
Upper extremity
46. Picks up a toy with the left hand, placed in front of the child, on the right-hand side.
47. Picks up a toy with the right hand, placed in front of the child, on the left-hand side.
48. Seated with his/her feet in front, he/she can touch a toy 45º behind him/her on the right.
49. Seated with his/her feet in front of him/her, he/she can touch a toy 45º behind on the left hand side.
50. Seated with the feet in front, he/she can touch toy in front of him/her and return to the initial position.
Transfers in WC
Transfers on a chair
Mobility and bed transfers
Mobility in the bathtub
Mobility indoors
Transport of objects
Outdoor Mobility
Functional mobility
Upper extremity
46. Picks up a toy with the left hand, placed in front of the child, on the right-hand side.
1. Sit the child down and place yourself in front of him/her, with your left hand holding an object (large and attractive), which is on the right-hand side of the child. Then place the object at an accessible distance from the child.
*As an aid, use objects that make a sound or movement of their own, and therefore attract his/her attention more.
2. Sit the child down and place an attractive object on a table to the right-hand side of the child.
*If the child has any mobility difficulty, make the movement as easy as possible. There is no need for the child to grasp the object, an attempt to grasp it or simply touching it would be valid.
3. Touch the child and place several objects, indicating to the child that he/she must take the object on his/her right, but with his/her left hand.
*If he/she can do so, try it standing up.
AR_CO_1
Communication and Language
Word comprehension
76. Moves towards sounds.
77. Understands "no"; recognizes her/his name or familiar names
78. Understands meaning when you talk about relationships between people and objects that are visible.
Understanding phrases
Functional use of communication
Communication and Language
Word comprehension
76. Moves towards sounds.
2. Observe if the baby or child moves his/her head towards the place where the sound is coming from.
3. Observe if the infant or child moves his/her head to look at the object.
4. *Always reinforce the child's attempts to perform the sound orientation action, no matter how small.
*Preferably, social reinforcement will be used, saying "very good, you have done very well" and these phrases will be accompanied by a smile.
AR_CO_1
Resolution of tasks in social contexts
Solving Problems
86. Tries to show you the problem or to communicate what needs to be done to solve the problem.
87. Must be helped immediately, if having a problem so that behavior will not be altered.
Information about yourself
Resolution of tasks in social contexts
Solving Problems
86. Tries to show you the problem or to communicate what needs to be done to solve the problem.
1. Place an object that the child wants or needs, such as a bottle of water, a glass of water, a doll he/she likes, etc., at a distance where the child cannot easily reach it.
2. Wait for the child to point to the object, then say "What do you want and name the object?
3. Reinforce* the child whenever he/she tries to imitate by vocalizing the name of the object and reinforce it when he/she does so, even if the production is not correct.
*Mainly make use of social reinforcement, such as "very good" "you did very well" and "you will reach the object".
AR_CO_1
Interactive and symbolic play
Interactive Play
91. Starts a play routine with which he/she is familiar
92. Participates in a simple play mindful of his/her turn
93. Tries to imitate the previous action of an adult during a play
Interaction with the peer group
Play with objects
Interactive and symbolic play
Interactive Play
91. Starts a play routine with which he/she is familiar
1. Place objects that are commonly found in the child's play routines that will first be toys that have a degree of similarity with the real object, progressively replacing them with small-scale toys, in order to enhance the development of the performance.
2. Then, the development of the game planning will be guided through questions such as "what is the teddy bear going to do now", "and later", etc.
3. Both verbal assistance and help with actions can be reduced as the game routine is structured around the dimensions of the symbolic game (decentration, substitution of objects, integration and planning).
4. The child's attempts to start the play routine, no matter how small, should always be reinforced. Social reinforcement will be used for this purpose.
AR_CO_1
Daily life routines
Guidance in time
101. She is generally aware of daily meal times and routines.
Homework
Daily life routines
Guidance in time
101. She is generally aware of daily meal times and routines.
2. Encourage the child to help set the table, first encourage him/her to help with simple actions, leave the cutlery on the table and letting the child lay the table.
3. At bath time, encourage the child to prepare the towels and clothes to be worn afterwards.
4. Orient the child from time to time by telling him/her what time it is. Use this time to teach the child about the time on the clock.
*Give the child a toy watch the clock hands of which he/she can move and tell the child to move the clock hands to the time that it is each time they are going to perform an action (eat, snack, bath or dinner among others).
AR_CO_1
Adaptive behavior
Self-harm behavior
104. Commits self-harm in habitual contextual situations.
105. Commits self-harm in situations of stress or anger for the child.
Heteroagressiveness (harming others)
Destruction of objects
Adaptive behavior
Self-harm behavior
104. Commits self-harm in habitual contextual situations.
A cognitive behavioural training programme will be implemented based on reinforcing positive behaviours and not reinforcing negative ones. If necessary, a token economy programme will be applied. A record of positive and negative behaviours will always be made in order to follow up on the child.

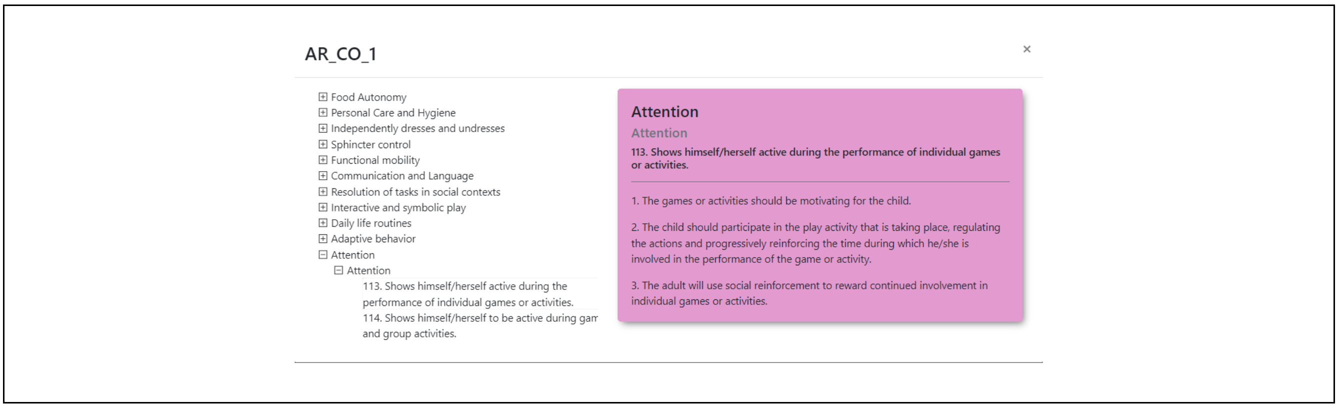

Figure S2. Example of profiling with the eEarlyCare-TP web application.

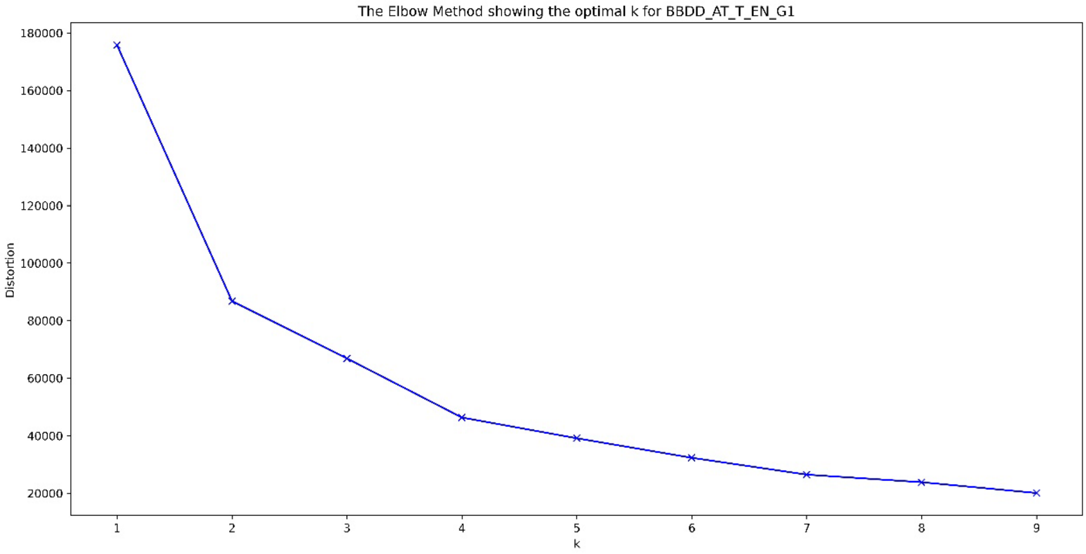

Figure S3. Elbow method in k-Means in Group 1.

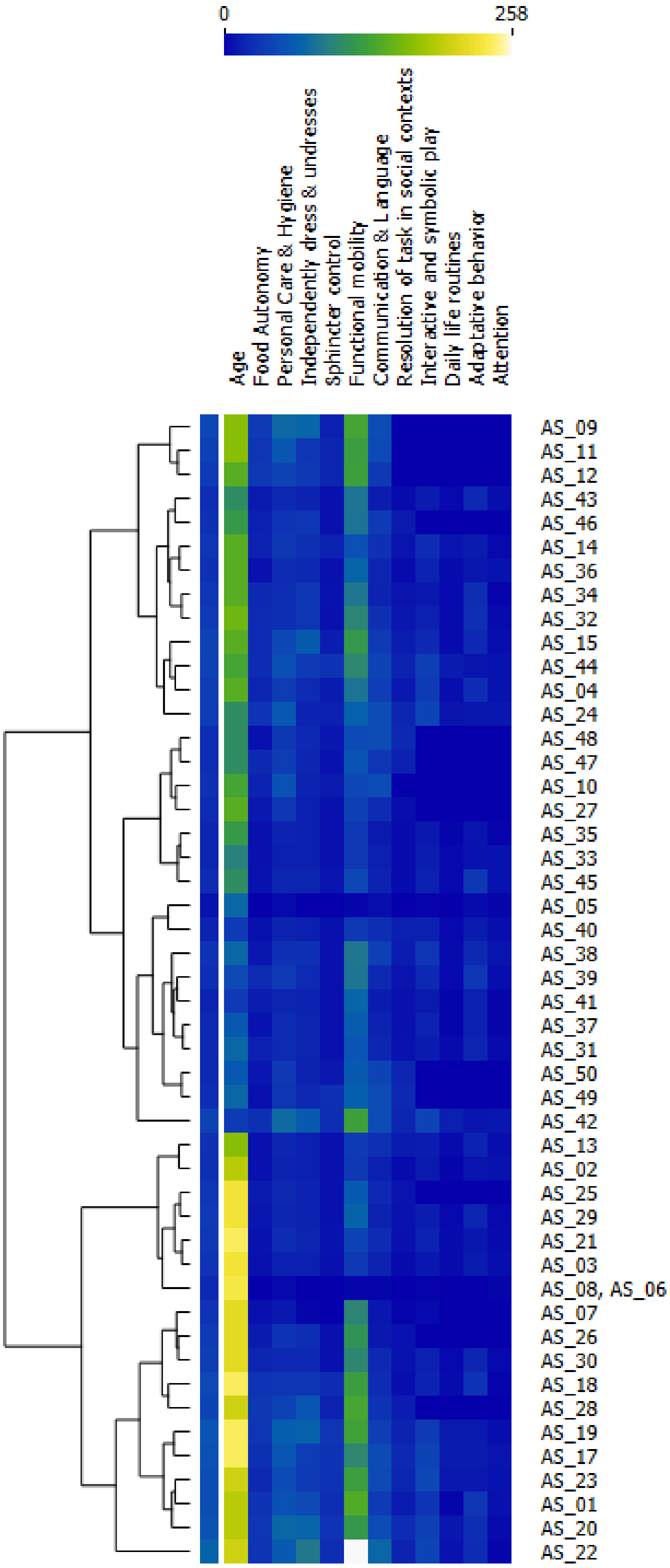

Figure S4. *k* Means patterns in Group 1.

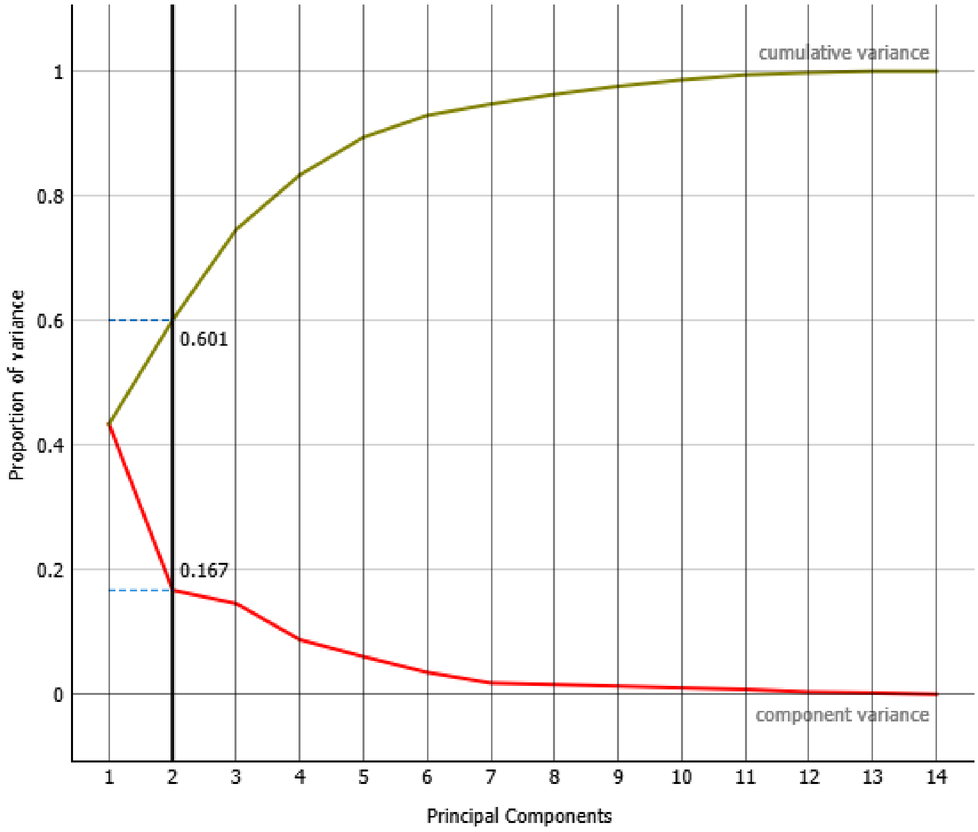

Figure S5. Principal Component Analysis in Group 1.

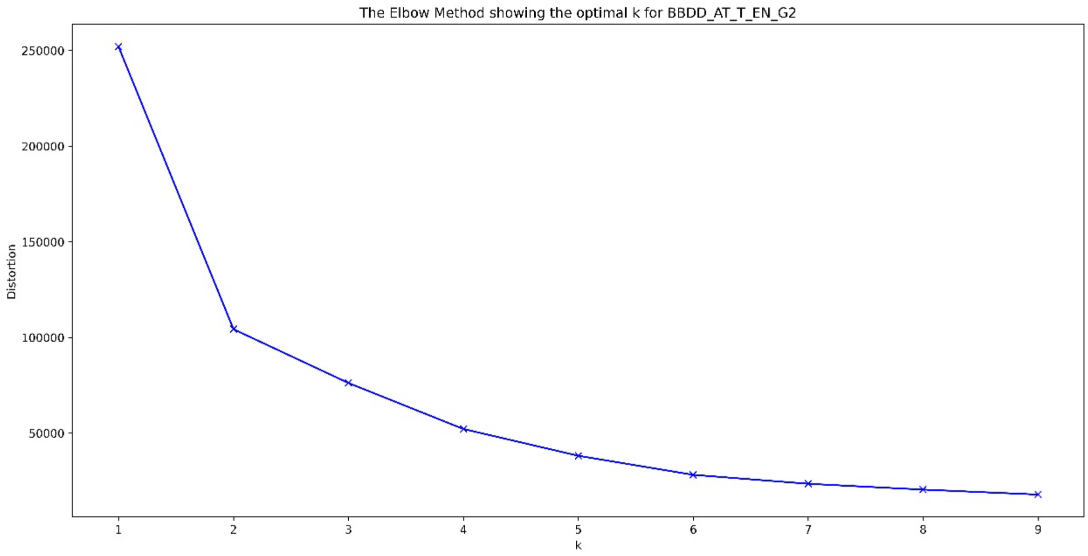

Figure S6. Elbow method in *k* Means in the Group 2.

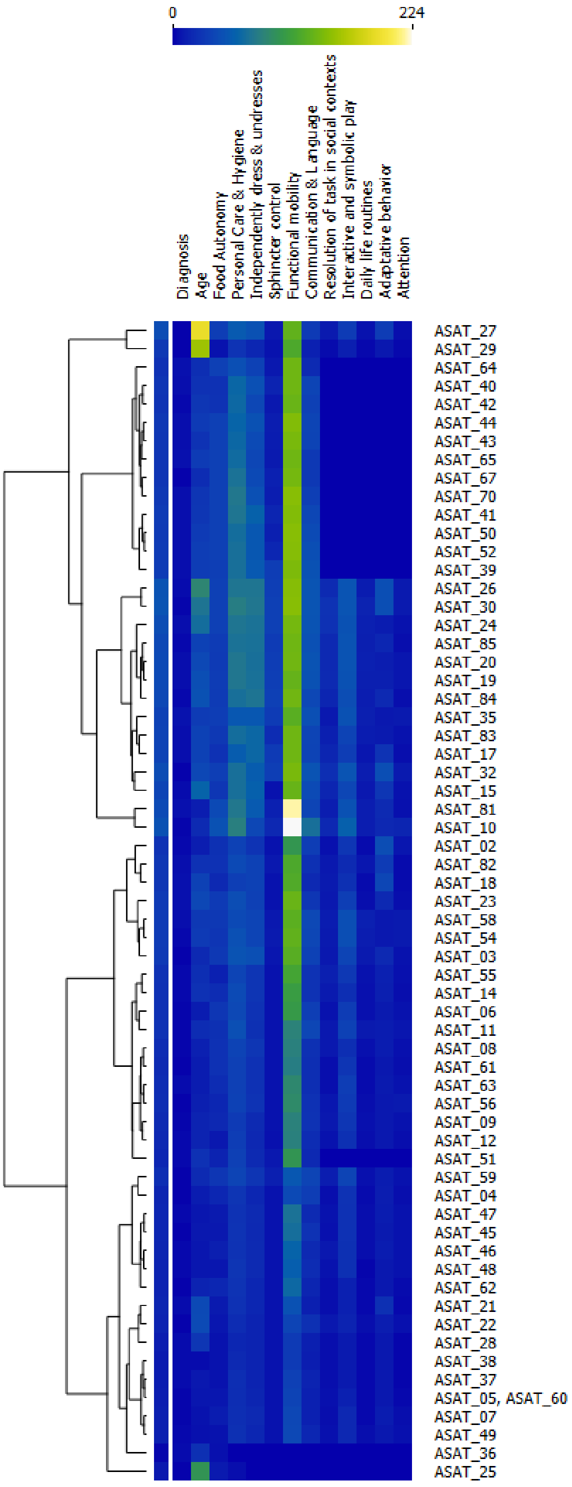

Figure S7. *k* Means patterns in Group 2.

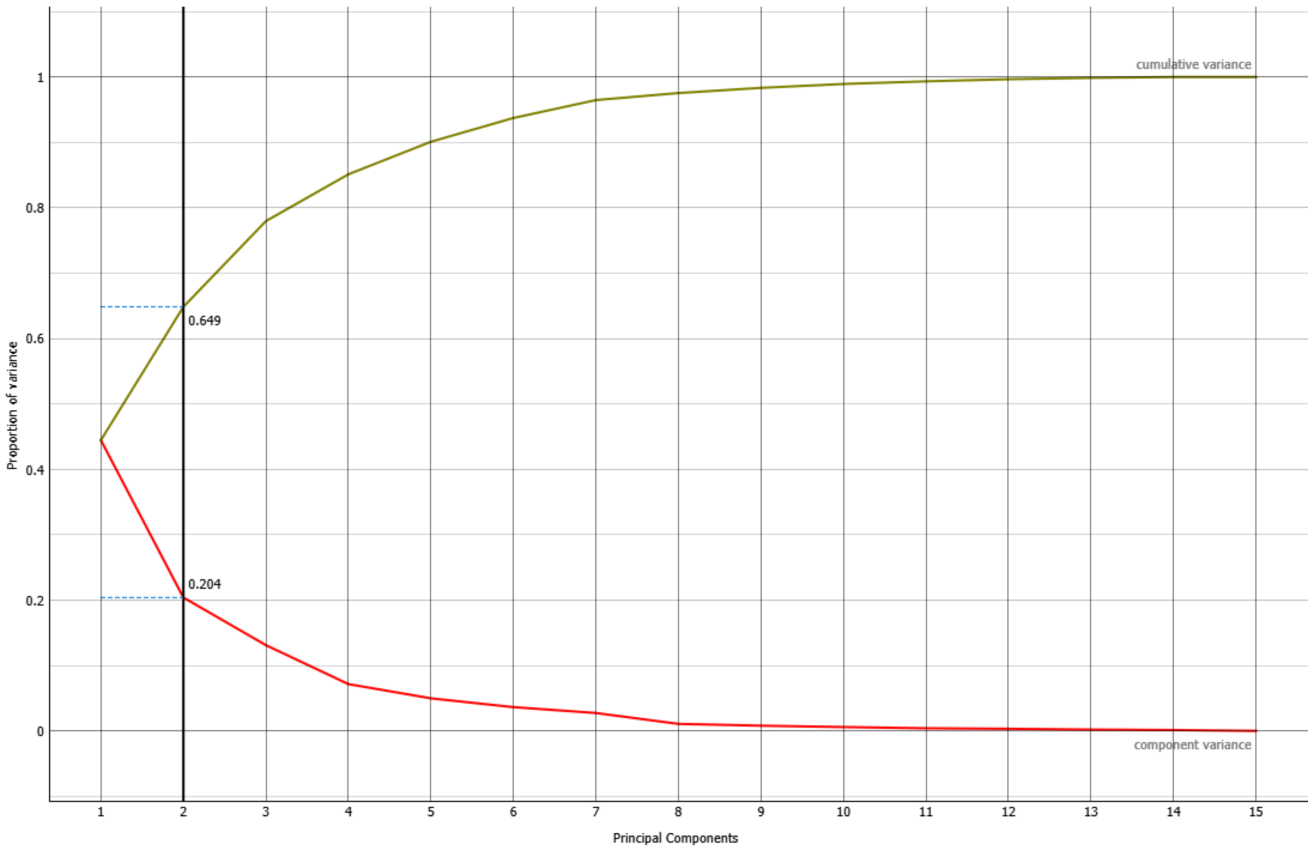

Figure S8. Principal Component Analysis in Group 2.
